# Targeted RNA-Seq profiling of splicing pattern in the *DMD* gene: exons are mostly constitutively spliced in human skeletal muscle

**DOI:** 10.1038/srep39094

**Published:** 2017-01-03

**Authors:** Anne-Laure Bougé, Eva Murauer, Emmanuelle Beyne, Julie Miro, Jessica Varilh, Magali Taulan, Michel Koenig, Mireille Claustres, Sylvie Tuffery-Giraud

**Affiliations:** 1 Université Montpellier, Laboratoire de Génétique de Maladies Rares, EA7402, Montpellier, F-34000, France; 2 Inserm U827, Montpellier, F-34000, France; 3 CHU Montpellier, Hôpital Arnaud de Villeneuve, Laboratoire de Génétique Moléculaire, Montpellier, F-34000, France

## Abstract

We have analysed the splicing pattern of the human Duchenne Muscular Dystrophy (*DMD*) NB transcript in normal skeletal muscle. To achieve depth of coverage required for the analysis of this lowly expressed gene in muscle, we designed a targeted RNA-Seq procedure that combines amplification of the full-length 11.3 kb *DMD* cDNA sequence and 454 sequencing technology. A high and uniform coverage of the cDNA sequence was obtained that allowed to draw up a reliable inventory of the physiological alternative splicing events in the muscular *DMD* transcript. In contrast to previous assumptions, we evidenced that most of the 79 *DMD* exons are constitutively spliced in skeletal muscle. Only a limited number of 12 alternative splicing events were identified, all present at a very low level. These include previously known exon skipping events but also newly described pseudoexon inclusions and alternative 3′ splice sites, of which one is the first functional NAGNAG splice site reported in the *DMD* gene. This study provides the first RNA-Seq-based reference of *DMD* splicing pattern in skeletal muscle and reports on an experimental procedure well suited to detect condition-specific differences in this low abundance transcript that may prove useful for diagnostic, research or RNA-based therapeutic applications.

Alternative splicing (AS) is a key mechanism for generating tissue or developmental stage-specific proteomic diversity in eukaryotes^1^,^2^. Muscle was one of the first tissues in which AS was widely observed in particular in contractile protein genes^3^. Recent global analyses of splicing programs have confirmed that skeletal muscle is among the tissues showing the largest number of tissue-specific AS events (ASEs)^4^.

The *DMD* gene encodes dystrophin, a cytoskeletal protein of 427 kDa accounting for only approximately 0.002% of the total striated muscle protein content but playing an essential role in muscle fiber integrity and function. Loss-of-function mutations cause Duchenne muscular dystrophy (DMD), the most common and severe form of progressive muscular dystrophy in children^5^. The *DMD* transcript can be categorized among the low abundance transcripts relative to other muscle transcripts. In adult skeletal muscle tissues, its concentration is estimated to be 5–10 molecules per nucleus as compared to 25,000–50,000 copies per nucleus for the highly abundant muscle transcript encoding the myosin heavy chain^6^. The *DMD* gene is remarkable by its length (2.2 Mb, the longest human gene) and genomic structure. About 99% of the gene is made of introns, some of them being very long introns exceeding 200 kb, while the translated coding sequence, which is fragmented into 79 exons, is only 11.3 kb. Seven independent tissue-specific promoters encode three full-length isoforms (including the Dp427m muscle one) and four N-terminally truncated proteins^5^.

There is no clear picture of the global splicing profile of the muscle transcript. Available data are not readily comparable because techniques of different sensitivity were used in previous studies that generally focused only on specific regions of the gene^7^,^8^,^9^,^10^. Considering the currently developed therapeutic approaches for DMD based on antisense oligonucleotide-mediated splicing modulation^11^, as well as the importance of splicing defects as a cause or a modifier of disease severity^12^,^13^, there is a clear need to elucidate the full splicing pattern of the *DMD* transcript.

Massively parallel RNA sequencing (RNA-Seq) has become a powerful technology to explore the complexity of mammalian transcriptomes^14^. Besides allowing for the comparison of gene expression changes in response to cellular differentiation, environmental factors or disease conditions, RNA-Seq can be used to accurately identify novel isoforms, assess relative transcript abundances and detect alternative exon and splice site usage in tissues or cells^15^. However, this approach has not yet provided information for all genes uniformly. Indeed a large fraction of sequence reads in RNA-Seq experiments are used up by highly expressed transcripts, thereby lowering the ability to detect other transcripts present at low levels^16^. This limitation is particularly detrimental to splicing analysis, which requires basically more input data than for gene expression analysis since a read must include the AS region to count towards splicing analysis^17^. Thus detection of alternative splicing within low abundance genes remains challenging. In this study, we designed a suitable strategy that relies on a *DMD*-targeted RNA-Seq protocol and the 454 technology to allow accurate and comprehensive inventory of physiological ASEs of the *DMD* transcript in human skeletal muscle.

## Results

### Design of an optimized RNA-Seq protocol for human *DMD* transcript analysis

**Figure 1:**
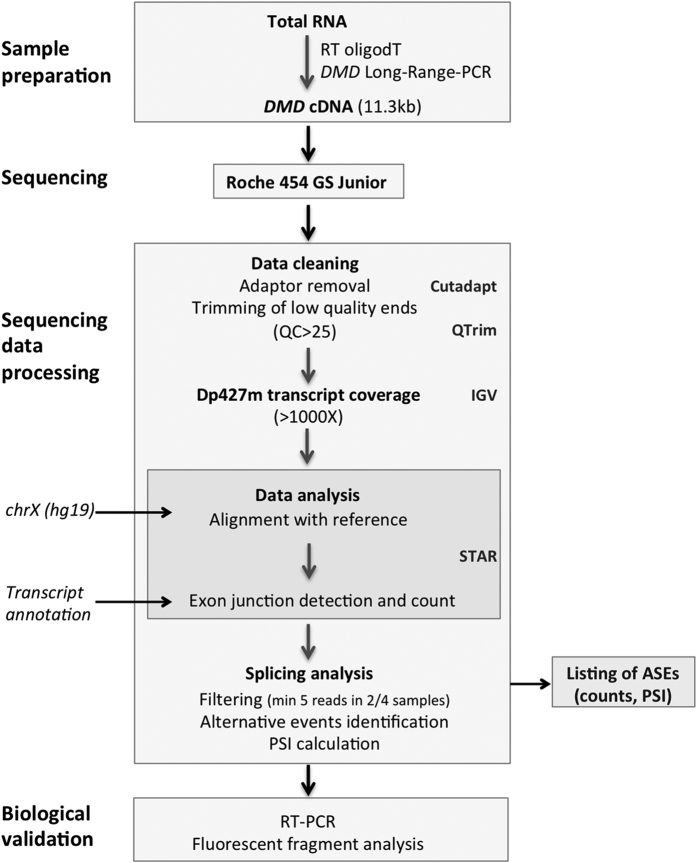
Experimental and analytical workflow for *DMD*-targeted RNA-Seq. Schematic depiction of the four-stage protocol developed for the analysis of the *DMD* gene splicing pattern by RNA-Seq including the sample preparation, sequencing, data analysis and biological validation. (RT) reverse transcription, (LR-PCR) Long Range-PCR, (QC) quality control, (IGV) Integrative Genome Viewer, (STAR) Spliced Transcripts Alignment to a Reference software, (PSI) Percent-Spliced-In, (ASEs) Alternative Splicing Events.

**Figure 2:**
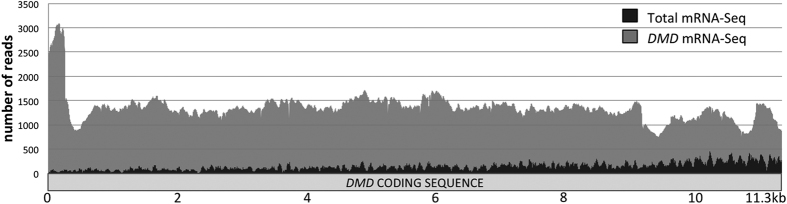
Comparison of *DMD* cDNA coverage in RNA-Seq experiments. Reads from each library were mapped to the *DMD* transcript (Dp427m mRNA, NM_004006.2) using Bowtie2 read aligner and *DMD* mRNA coverage was visualized through the Integrative Genome Viewer (IGV). Shown are the IGV coverage plots from (dark grey) publicly available total mRNA-Seq data (Illumina Human Body Map project 2.0, skeletal muscle tissue, GEO sample GSM759515, 2 × 50 bp paired end, 8.2 G bases), and (light grey) *DMD* mRNA-Seq data generated in this study (skeletal muscle sample n°20316, Myobank).

Before designing our experimental workflow, a preliminary analysis of RNA sequencing data of human skeletal muscle tissue available in the Illumina’s Body Map (IBM) transcriptome project^18^ had shown us that the read depth along the *DMD* transcript was globally insufficient (50X–200X) despite the huge amount of reads produced, to allow reliable detection of ASEs present at low level. This observation prompted us to set up a *DMD*-targeted RNA-Seq approach (Fig. 1) that combines the amplification of the full *DMD* coding sequence (11.3 kb) with the Roche 454 sequencing technology. Typically 454 reads are >300 bp long and are likely to span more than two exon junctions that helps to identify non-canonical splicing events. To assess the performance of our procedure, a first comparison has been made between the data obtained from the analysis of one skeletal muscle sample using our targeted approach (average read length: 387 bp, 15.8 Mb) and the data from the IBM project (human skeletal muscle RNA GSM759515, 2 × 50 bp paired end, 8.2 Gb). Visualization of the aligned reads to the *DMD* Dp427m isoform showed differences in *DMD* mRNA sequencing depth and coverage (Fig. 2). With the total mRNA sequencing technology, the mean depth per base was 142 (+/−82 SD) reads and a marked decrease of coverage at the 5′end of the transcript was noticed (about 1 kb is covered by less than 50 reads) due to a bias toward the identification of sequences from the 3′ end of *DMD* mRNA as previously described^19^. By using our *DMD*-targeted approach, a relatively uniform coverage was obtained with a mean depth of 1,363 (+/−302 SD) reads per base that would theoretically allow ASEs as low as 1% to be reliably detected (>10 reads).

The Spliced Transcripts Alignment to a Reference software (STAR), specially designed for aligning long reads^20^, was then used to identify *DMD* canonical splice junctions as well as potential new ones from junction-spanning reads. Compared to TopHat2, a popular mapper suitable for processing relatively short reads, STAR performed better both in the total mRNA-Seq and our *DMD*-targeted mRNA-Seq datasets (Table 1) for the positioning of the 78 canonical junctions and the discovery of alternative splice junctions as illustrated with pseudoexon 1a, an already known case of pseudoexon insertion in the *DMD* transcript^21^.

### Splicing pattern of *DMD* transcript in skeletal muscle tissue

**Figure 3:**
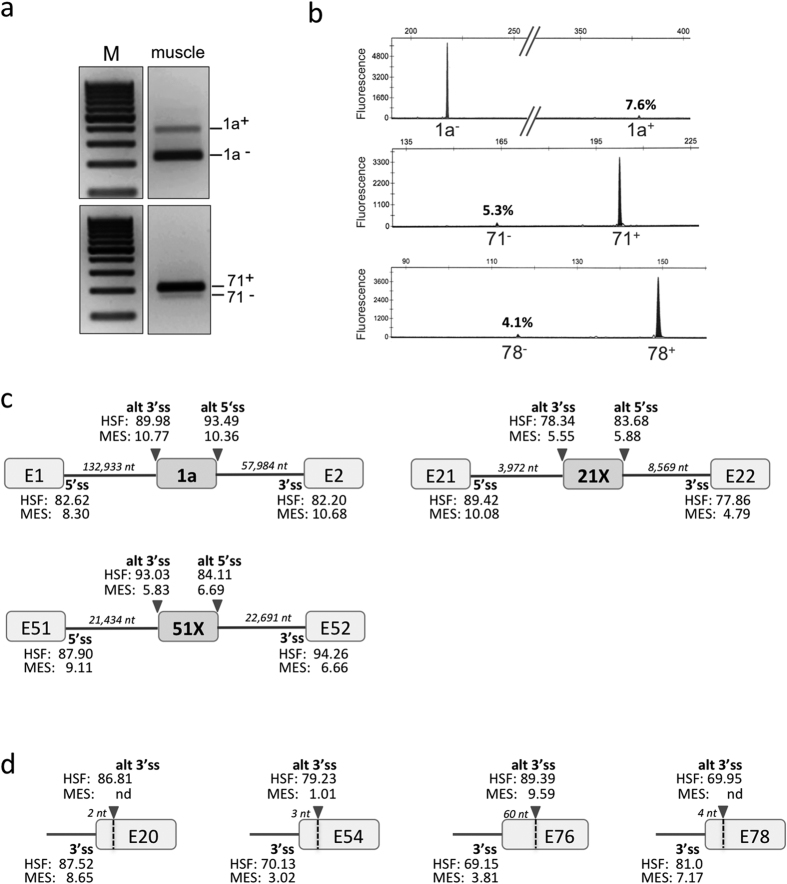
Biological validation of Alternative Splicing Events in skeletal muscle. Examples of RT-PCR validations of the three most represented ASEs by (**a**) standard RT-PCR and agarose gel electrophoresis or (**b**) capillary electrophoresis analysis of fluorescent PCR products. M, molecular-weight size marker. Quantification data (mean +/−SD) from at least three independent experiments are reported in Table 3. (**c**) Splice-site strength of pseudoexons. MaxEntScan (MES) and Human Splicing Finder (HSF) scores are given for alternative 3′ and 5′ splice sites (alt 3′ss, alt 5′ss) of pseudoexons 1a, 21X and 51X and for splice sites of adjacent constitutive exons (3′ss, 5′ss). The size of the intronic regions (in nucleotides, nt) flanking the pseudoexon is indicated in italics. (**d**) Splice-site strength of alternative 3′ splice sites. MES and HSF scores are given for exonic alternative 3′ splice sites (alt 3′ss) and adjacent authentic 3′ splice sites (3′ss). nd, not detected. The vertical dotted lines mark the position of the alternative 3′ss in exons 20, 54, 76 and 78. The number of deleted nucleotides (nt, in italics) in the mature dystrophin transcript when the alternative 3′ss is used is indicated on top of each exon.

Four independent skeletal muscle tissue samples from normal young men were used to establish the splicing pattern of the muscular *DMD* transcript. We applied our established *DMD*-targeted RNA-Seq protocol, and the splice junction reads obtained both for the 78 canonical junctions and novel splice junctions in the 4 samples are detailed in the Supplementary Table S1. Among the non-canonical splice junctions detected, we chose to consider only those consistently detected with a minimum of 5 read counts in at least 2 out of the 4 muscle samples analysed for further analyses. Strikingly only 12 ASEs could be identified (Table 2), which can be divided into three different categories: exon skipping (n = 5), cryptic exon inclusion (n = 3) and 3′ alternative splice site (ss) usage (n = 4). Most of them (9/12) are low level ASEs (≤1%) and the three most represented events (del71, del78, PE1a) do not exceed 3%. It is noteworthy that the majority of them (66%, 8/12) preserve an open reading frame. While some events have already been reported (del9, del71, del78, PE1a), the other ones are here described for the first time (Supplementary Table S2). The very low level of detected ASEs precluded their experimental validation by RT-PCR in most cases, but we were able to conduct these analyses for three of them (del71, del78, and PE1a). We performed either standard or fluorescent RT-PCR using primers that amplify both the included and the skipped isoforms, and analysed PCR products by gel electrophoresis or fragment analysis on a capillary sequencer, respectively. The three ASEs were successfully validated (Fig. 3), with a greater concordance between RNA-Seq and fragment analysis data (Table 3).

In addition to exon skipping events, the RNA-Seq analysis has revealed the usage of alternative splice sites located either at proximity of the natural ones or deep in the introns (Table 2). The reliability of the bioinformatics analysis for these new sites was systematically verified by inspection of the raw sequencing reads. In particular for pseudoexons, we verified that the whole sequence of the pseudoexon could be retrieved from a single read. Erroneously annotated alternative splice sites were found at the exons 3/4 and exons 14/15 junctions due to the presence of homopolymers and/or sequence homologies that led to misalignment (Supplementary Table S1). These alternative junctions were thus excluded from our analysis. With the exception of the PE1a, the 6 other reported events (2 pseudoexons, and 4 alternative 3′ ss) are newly described in this study (Table 2). The strenght of these alternative splice sites was evaluated by maximum entropy scores (MaxEntScan, MES) and the Human Splicing Finder (HSF) program (Fig. 3).

All three pseudoexons displayed high 5′ and 3′ splice site scores, thus supporting their use to some extent in the *DMD* transcripts. Regarding the detected 3′ alternative ss, all are exonic and their calculated scores were lower than the scores of the adjacent natural splices in all cases except for the exon 76 + 59 alternative 3′ss, which exhibits scores (HSF: 89.39 and MES: 9.59) significantly higher than the natural 3′ss of exon 76 (HSF: 69.15 and MES: 3.81) (Fig. 3). When used, a new transcript is produced carrying an in-frame deletion of 20 amino acids (Pro3600 to Gln3619) in the C-terminal domain of dystrophin that is not predicted to alter a protein-protein interaction domain. The RNA-Seq analysis also disclosed splicing at a previously undescribed NAGNAG tandem site in *DMD* exon 54 that deletes one amino acid (p.Glu2625). This novel junction was reliably detected in all four analysed skeletal muscle samples (11–28 junction reads).

Lastly, we have drawn up a list of all detected exon skipping events but that failed to reach the minimum criteria set (*i.e.* a minimum of 5 reads in 2 out of the 4 samples analysed) (Supplementary Table S1). Interestingly we noticed that half of them (13/25, 52%) are located in the contiguous block of the 20 in-frame exons, encoding portions of the central rod domain of dystrophin, and the most represented ones (del25, del28, del28 + 29, del38) match with exons frequently involved in nonsense-induced exon skipping events that contribute to modify disease severity in patients. This observation further supports the idea that these exons have peculiar features regarding splicing, which may make them much more prone to exon skipping when mutated in patients^22^.

## Discussion

Over half of the multi-exonic human genes are believed to have splice variants^23^. Due to its large number of exons, the *DMD* gene was *a priori* a good candidate for the occurrence of multiple alternative splicing events. A literature survey supports this common assumption with many reports describing alternative splicing events in the *DMD* gene since its discovery in 1986 (Supplementary Table S2). However these data derive from studies that are very heterogeneous in terms of the techniques used (northern blot, PCR, nested PCR), specificity of the transcript isoform analysed (Dp427m and/or other isoforms), biological samples analysed (human *versus* animal tissues, skeletal muscle *versus* other tissues or lymphocytes, patients *versus* normal controls), and gene regions explored (most studies focused on specific regions), that makes qualitative and quantitative comparisons between these different datasets difficult. A comprehensive and unbiased inventory of the *DMD* splicing events in skeletal muscle was lacking.

The recently developed deep-sequencing technologies allows a far more precise quantification of transcript levels and their isoforms than other methods^14^. In this study, a targeted RNA-Seq procedure was chosen to increase the sequencing depth and therefore to allow reliable detection of alternative splice junctions in the *DMD* gene. The deep RNA sequencing strategy was reasonably expected to expand the repertoire of ASEs in the *DMD* gene. Yet surprisingly, the vast majority of the 79 *DMD* exons were found to be constitutively spliced in skeletal muscle under physiological conditions. Hence, our results would indicate that the frequency of ASEs previously reported in the *DMD* muscular transcript may have been overestimated, possibly due to methodological considerations.

Exon-skipping was the most common form of the ASEs detected (5/12, 41.7%). Notwithstanding the large number of exons in the *DMD* gene, skipping of only a single or a limited number of exons were characterized (Table 2 and Supplemental Table 1). Skipping of long stretches of exons were not found in normal human skeletal muscle contrary to what can be observed in pathological condition, where multiple exon skipping rearrangements have been reported to occur, notably in revertant fibers to restore dystrophin expression in patients^24^ or in the mdx model^25^. Though few in number, the identity of the identified ASEs in this study provides strong support for the reliability of our RNA-Seq analysis. Indeed, several of them correspond to previously described ASEs occurring in a tissue-specific or developmental stage-specific manner in the full-length muscular transcript or in other isoforms^9^,^26^,^27^. In particular, exon 71 and exon 78 are alternatively spliced in Dp71, one of the shortest dystrophin isoform and the major *DMD* gene product in many non-muscle tissues. *DMD* transcripts alternatively spliced at the 3′ end encode functionally distinct protein isoforms that are likely to remodel protein-protein interaction networks^28^ in particular with components of the dystrophin-glycoprotein complex (DGC). The in-frame skipping of exon 71 results in loss of the syntrophin-binding site from the protein^29^, while the absence of exon 78 causes a frameshift that replaces the 13 C-terminal dystrophin amino acids residues with 31 new ones defining a protein with a novel hydrophobic carboxy terminus^30^. Exon 78 is spliced out in the embryonic muscle dystrophin isoform. This developmentally regulated alternative splicing is highly conserved throughout vertebrates, arguing for a critical functional role of this longer C-terminal protein domain during the embryonic stage of development. By contrast, dystrophin exon 78 is required for muscle structure maintenance in adult skeletal muscle. Its abnormal exclusion in patients suffering from myotonic dystrophy (DM) due to depletion of the MBNL1 splicing factor likely contributes to the progressive dystrophic process in DM type1 patients^31^. In accordance with these data, exon 78 was found to be included in about 98% of the muscular transcripts in our RNA-Seq experiments as was exon 71. The traces of exon skipping detected in muscle may reflect the complexity of the mechanisms controlling the inclusion of these two exons in a tissue-specific or developmental stage-specific manner^9^,^32^. It is noteworthy that *DMD* exon 71 and exon 78 are very short exons (39 and 32 bp, respectively) that share some characteristics with the recently described microexons in neurons, a set of highly conserved short exons, which are strongly regulated by RNA-binding proteins (RBPs) and functionally modulate tissue-dependent protein-protein networks^33^,^34^.

The RNA-Seq analysis has revealed the usage of alternative splice sites located either in the close vicinity of the natural sites or deep in the introns (Table 2), which are all newly described except pseudoexon 1a. The pseudoexon 1a originating from intron 1 was initially identified in lymphocytes^21^, where it is included in more than 50% of the illegitimate *DMD* transcripts. The inclusion of this out-of-frame extra-exon may represent a post-transcriptional control in cells that normally do not express the dystrophin protein (like lymphocytes), while a low inclusion rate is observed in skeletal muscle transcripts (3%). Due to the size of the *DMD* introns, multistep splicing events (recursive or nested splicing) are frequently used to splice out a single intron^35^. Interestingly, the 5′ and 3′ genomic coordinates of the identified pseudoexons in our study (1a, 21X, 51X) are similar to 6 out of the 145 biocomputational predicted positions associated with either 5′ or 3′ recursive splicing of multi-step intron removal recently identified by capture-pre-mRNA-seq of intermediately spliced dystrophin transcript^35^. Whether these 6 positions represent true motifs required for 5′and 3′ recursive splicing remains to be experimentally demonstrated, but our findings show that they can be used in combination for the inclusion of pseudoexons in the mature transcripts.

As previously reported in other human genes^36^, we observed that alternative acceptors were the second most common (4/12, 33%) type of AS detected in *DMD* after exon skipping. All are exonic alternative 3′ splice sites that induce partial exon deletion when used, and the major one involves a NAGNAG tandem site in *DMD* exon 54. NAGNAG motif occurs in 30% of human genes and appears to be functional in at least 5% of human genes^37^,^38^. Their use results in the production of the two distinct isoforms distinguishable by three nucleotides (NAG). These subtle changes may nonetheless be of functional relevance by changing local hydrophobicity and charge, varying the distances between relevant sites in proteins or changing recognition sequences for post-translational modifications. This is the first functional NAGNAG motif identified in the *DMD* gene. Splicing at this site deletes the polar amino acid glutamine at position 2625 of dystrophin repeat 21 which forms part of the region involved in specific protein/lipid interactions that favours homogeneous dystrophin distribution along the membrane^39^. Inspection of the 77 remaining 3′ss did not reveal any other NAGNAG tandem acceptor motifs in the *DMD* gene.

In conclusion, this study provides the first RNA-Seq-based reference of *DMD* splicing pattern in normal muscle. The strategy used allows the analysis of the whole 11.3 kb-cDNA sequence all at once that may prove useful for mutational analysis or monitoring the impact of splicing interventions on transcript structure in patients with Duchenne muscular dystrophy. Undesirable alternative splicing events may impact the outcome of exon skipping or *trans*-splicing approaches. Unlike other isoforms^26^,^27^,^40^, expression of the *DMD* gene is tightly regulated in skeletal muscle to preserve the production of a full-length transcript and a 427 kDa dystrophin protein, even if some protein domains are considered to be functionally dispensable^41^. The *DMD* gene represents a paradigm for extreme splicing conditions that illustrates perfectly how accurate the process of splice site selection shoud be to include the 79 exons into the mature transcript concomitantly with the repression of near perfect pseudoexons or cryptic 3′ss activation. A large piece of work remains to be done to elucidate the splicing regulation in this huge gene, which will contribute to reveal new insights into basic splicing mechanisms.

## Methods

### Muscle samples

Two striated muscle tissue samples (n°20316 and n°27563) from healthy male individuals (aged 29 and 19, respectively) were obtained from the Myobank-AFM (Paris, France). Two additional control skeletal muscle total RNA samples from 20 year-old males were purchased from Clontech (ref #636534, lots #1404229A and #1406360A).

### RNA extraction and cDNA synthesis

Total RNA was extracted from biopsies using the SV total RNA extraction kit (Promega) and RNA integrity was checked using the Agilent RNA 6000 Nano kit with Agilent 2100 bioanalyzer (Agilent Technologies). 700 ng of total RNA were used to synthesize template cDNA by reverse transcription with the Superscript^®^ II (Thermo Scientific) and oligodT primers for RNA-Seq experiments.

### Long Range (LR)-PCR

One fifth of the RT product was used as template to amplify the full-length *DMD* cDNA as a single long fragment (11.3 kb) by using the GoTaq^®^ Long PCR Master Mix (Promega) and primers located in exon 1 of the muscle isoform (Dp427m) (5′-CTTTCCCCCTACAGGACTCAG-3′) and in the 3′UTR (5′-CCAAATCATCTGCCATGTGG-3′). Cycle parameters were programmed as 94 °C for 2 min, followed by the first 10 cycles of 93 °C for 15 s, 58 °C for 30 s, and 68 °C for 11 min and a 20 s auto-extension of elongation time for cycles 11–35. After verification of amplicon size by agarose gel electrophoresis, LR-PCR reactions were purified using Qiaquick PCR purification kit (Qiagen) and quantified using the NanoDrop 2000 spectrophotometer (Thermo Scientific).

### Construction of 454 libraries and sequencing

For each library, around 1 μg of purified PCR product were fragmented by nebulization (2.1 bars of nitrogen for 1 min) and subjected to library preparation with the Rapid Library Preparation Kit (Roche) according to manufacturer’s instructions. The different samples were labeled via ligation of Multiplex Identifiers (MID) oligonucleotide adaptors to allow multiplexing. Three MID-containing libraries were quantified using the Qubit^®^ Fluorometer and pooled in equimolar amounts into a single sample prior to emulsion PCR amplification and sequencing in parallel on the Roche GS Junior 454 sequencer giving a mean total number of 69,060 reads (387 bp mean length) per library. The data obtained were then sorted according to their tag sequences.

### Bioinformatics

Raw reads were edited and filtered prior to analysis. A dedicated analysis pipeline was developed using the Galaxy framework (http://galaxyproject.org)^42^. First, relevant adapter sequences were removed with Cutadapt (v.1.3, default parameters), and quality-based trimming at the 3′ ends of reads was performed using the Qtrim tool (v.1.1, parameters: mean quality = 25, window size = 20, minimum read length = 40 nt). Cleaned reads were then mapped to the Human X chromosome reference sequence (hg19, UCSC) with STAR (v. 2.3, annotations from UCSC, parameters: -sjdbOverhang 29 -outFilterMismatchNoverLmax 0.05 -outSJfilterReads Unique, outFilterMultimapNmax 1)^20^. A script developed in-house was developed to annotate identified splice junctions, which can be obtained upon request. The output data is processed to obtain *DMD* mRNA coverage, as well as a list of identified splice junctions and their counts. Only new junctions covered by a minimum of 5 reads in at least two out of the four biological replicates were considered for further analysis. The Percent-Spliced-In (PSI) was calculated for each splicing event using intron-centric metrics^43^, with the SJPIPE pipeline, from the ipsa package (parameters: margin = 0, deltaSS = 0, mincount = 0, https://github.com/pervouchine/ipsa). For the comparison of the *DMD*-targeted versus publicly available total mRNA sequencing data from the Illumina Human Body Map project 2.0 (skeletal muscle tissue, 2 × 50 bp, GEO sample GSM759515), raw reads were mapped to the Human X chromosome sequence with Bowtie2 (v2.default parameters)^44^ and exon junctions detection and count was performed by TopHat2^45^ (v2.0.2, Gene Model Annotations option: chrX_GTF downloaded from UCSC). Two different splice site prediction algorithms were used for computational scoring of 5′ and 3′ splice sites: the Human Splicing Finder tool (http://www.umd.be/HSF3/), which uses the weight matrix model^46^ and the MaxEntScan (MES) (http://genes.mit.edu/burgelab/maxent/Xmaxentscan_scoreseq_acc.html) based on the maximum entropy principle^47^.

### Experimental validation of Alternative Splicing Events

Independent RT experiments were performed by using random primers for experimental validation. 1.5 μL of cDNA was used as template for PCR amplification in a 25 μL total volume with the Taq DNA Polymerase (New Englands Biolabs) and primers hybridizing to upstream and downstream exons (sequences available upon request). For conventional RT-PCR, the 30 cycles-amplified products were separated on 1.8% agarose gels and spliced products were quantified with the Quantity One (v.4.6.9) software (Bio-Rad). For semi-quantitative PCR, fluorescein-labeled forward primers were used and 26 cycles were done. Capillary electrophoresis analysis was performed using 1 μl of PCR product added to 18 μl of formamide and 0.5 μl of ROX 400 HD fluorescent size standards (Applied Biosystems). Amplified products were separated on an ABI 3130 XL DNA analyzer and the peaks areas were measured by the GeneMapper v4.0 software. Ratios of splicing isoforms were determined as the peak area of one specific isoform divided by the total peak areas for the two or three detected isoforms. Data represent the mean +/−SD of at least three independent assays.

## Additional Information

**How to cite this article**: Bougé, A.-L. *et al*. Targeted RNA-Seq profiling of splicing pattern in the *DMD* gene: exons are mostly constitutively spliced in human skeletal muscle. *Sci. Rep.* **7**, 39094; doi: 10.1038/srep39094 (2017).

**Publisher's note:** Springer Nature remains neutral with regard to jurisdictional claims in published maps and institutional affiliations.

## Supplementary Material

Supplementary Information

Supplementary Table S1

## Figures and Tables

**Table 1:** Performance of TopHat2 and STAR spliced aligners on Total mRNA-seq and *DMD* mRNA-seq data for the detection of exon junctions in the *DMD* transcript.

	TopHat2		STAR	
	Total mRNA-seq	*DMD* mRNA-seq	Total mRNA-seq	*DMD* mRNA-seq
exon junctions: all	78	93	98	128
exon junctions: canonical	73	78	78	78
canonical junctions mean coverage (+/−SD)	142 (+/−84)	697 (+/−134)	117 (+/−69)	1144 (+/−254)
PE1a junctions mean coverage	0	5	0	97

“Exon junctions: all” refers to the number of detected junctions in the RNA-seq datasets including both canonical (n = 78 in the *DMD* gene) and new junctions due to alternative splicing events. Mean coverages given by TopHat2 and STAR for canonical junctions and for pseudoexon 1a (PE1a), a well-documented example of pseudoexon insertion in the mature muscular *DMD* transcript, are indicated.

**Table 2:** List of ASEs identified by *DMD* targeted RNA-Seq in skeletal muscle tissue.

Event name	Muscle mean ASE (%)	Predicted reading frame
del9	0.63	in frame
**del71**	**1.80**	**in frame**
del73	0.32	in frame
del74	0.49	in frame
**del78**	**2.48**	**out of frame/elongated C-term**
**PE1a** (162 nt)	**3.06**	**out of frame**
PE21X (66 nt)	0.40	in frame
PE51X (84 nt)	0.21	in frame
3′ss exon 20 (−2 nt)	0.18	out of frame
3′ss exon 54 (−3 nt)	1.12	in frame
3′ss exon 76 (−60 nt)	0.33	in frame
3′ss exon 78 (−4 nt)	0.43	out of frame

The three major ASEs detected in skeletal muscle are indicated in bold. *DMD* exon 78 skipping changes the reading frame and replaces the 13 C-terminal aminoacids of dystrophin by 31 new amino acids (elongated C-terminus). The size of the inserted pseudoexons (PE) in the mature dystrophin transcripts is indicated in brackets (nt, nucleotides). The number of exonic nucleotides (nt) deleted due to the use of alternative 3′ splice sites (3′ss) is indicated in brackets. ASE(%) = (1 − PSI) × 100 for exon skipping and alternative 3′ss, and ASE(%) = PSI × 100 for pseudoexon (PE) inclusion.

**Table 3:** **E**xperimental validation by independent techniques of ASEs identified by RNA-Seq.

	RNA-seq	Gel analysis	Fragment analysis
PE1a	3.06 (+/−1.00)	12.2 (+/−2.14)	7.56 (+/−3.55)
del71	1.81 (+/−0.67)	7.20 (+/−1.63)	5.28 (+/−0.64)
del78	2.48 (+/−0.57)	nd	4.10 (+/−0.30)

ASEs quantification using either RNA-seq, gel or fragment analysis techniques as presented in Fig. 3. ASE(%) = (1 − PSI) × 100 for exon skipping and alternative 3′ss, and ASE(%) = PSI × 100 for pseudoexon 1a (PE1a) inclusion. The means (±SD) of at least three independent experiments are given. nd, not detected.

## References

[b1] Pan Q., Shai O., Lee L. J., Frey B. J. & Blencowe B. J. Deep surveying of alternative splicing complexity in the human transcriptome by high-throughput sequencing. Nat. Genet. 40, 1413–1415 (2008). 18978789 10.1038/ng.259

[b2] Raj B. & Blencowe B. J. Alternative Splicing in the Mammalian Nervous System: Recent Insights into Mechanisms and Functional Roles. Neuron 87, 14–27 (2015). 26139367 10.1016/j.neuron.2015.05.004

[b3] Llorian M. & Smith C. W. J. Decoding muscle alternative splicing. Curr. Opin. Genet. Dev. 21, 380–7 (2011). 21514141 10.1016/j.gde.2011.03.006

[b4] Castle J. C. . Expression of 24,426 human alternative splicing events and predicted cis regulation in 48 tissues and cell lines. Nat. Genet. 40, 1416–1425 (2008). 18978788 10.1038/ng.264 PMC3197713

[b5] Muntoni F., Torelli S. & Ferlini A. Review Dystrophin and mutations: one gene. several proteins, multiple phenotypes. Lancet, 44, 731–740 (2003). 10.1016/s1474-4422(03)00585-4 14636778

[b6] Tennyson C. N., Shi Q. & Worton R. G. Stability of the human dystrophin transcript in muscle. Nucleic Acids Res. 24, 3059–3064 (1996). 8760894 10.1093/nar/24.15.3059 PMC146056

[b7] Surono A., Takeshima Y., Wibawa T., Pramono Z. A. & Matsuo M. Six novel transcripts that remove a huge intron ranging from 250 to 800 kb are produced by alternative splicing of the 5′ region of the dystrophin gene in human skeletal muscle. Biochem. Biophys. Res. Commun. 239, 895–899 (1997). 9367866 10.1006/bbrc.1997.7579

[b8] Sironi M. . The dystrophin gene is alternatively spliced throughout its coding sequence. FEBS Lett. 517, 163–166 (2002). 12062429 10.1016/s0014-5793(02)02613-3

[b9] Feener C. A., Koenig M. & Kunkel M. Alternative splicing of human dystrophin mRNA generates isoforms at the carboxy terminus. Nature, 338, 509–511 (1989). 2648158 10.1038/338509a0

[b10] Zhang Z. . Identification of seven novel cryptic exons embedded in the dystrophin gene and characterization of 14 cryptic dystrophin exons. J. Hum. Genet. 52, 607–17 (2007). 17579806 10.1007/s10038-007-0163-0

[b11] Guiraud S., Chen H., Burns D. T. & Davies K. E. Advances in genetic therapeutic strategies for Duchenne muscular dystrophy. Exp. Physiol. 100, 1458–1467 (2015). 26140505 10.1113/EP085308 PMC4973818

[b12] Miro J. . FUBP1: a new protagonist in splicing regulation of the DMD gene. Nucleic Acids Res. 43, 2378–2389 (2015). 25662218 10.1093/nar/gkv086 PMC4344520

[b13] Disset A. . An exon skipping-associated nonsense mutation in the dystrophin gene uncovers a complex interplay between multiple antagonistic splicing elements. Hum. Mol. Genet. 15, 999–1013 (2006). 16461336 10.1093/hmg/ddl015

[b14] Wang Z., Gerstein M. & Snyder M. RNA-Seq: a revolutionary tool for transcriptomics. Nat. Rev. Genet. 10, 57–63 (2009). 19015660 10.1038/nrg2484 PMC2949280

[b15] Anders S., Reyes A. & Huber W. Detecting differential usage of exons from RNA-seq data. Genome Res. 22, 2008–2017 (2012). 22722343 10.1101/gr.133744.111 PMC3460195

[b16] Halvardson J., Zaghlool A. & Feuk L. Exome RNA sequencing reveals rare and novel alternative transcripts. Nucleic Acids Res. 41, 1–10 (2013). 22941640 10.1093/nar/gks816 PMC3592422

[b17] Hooper J. E. A survey of software for genome-wide discovery of differential splicing in RNA-Seq data. Hum. Genomics 8, 3 (2014). 24447644 10.1186/1479-7364-8-3 PMC3903050

[b18] Florea L., Song L. & Salzberg S. L. Thousands of exon skipping events differentiate among splicing patterns in sixteen human tissues. F1000Research, 2, 188 (2013). 24555089 10.12688/f1000research.2-188.v1 PMC3892928

[b19] Nagalakshmi U., Waern K. & Snyder M. RNA-Seq: A Method for Comprehensive Transcriptome Analysis. Curr. Protoc. Mol. Biol. 4, 1–13 (2010). 10.1002/0471142727.mb0411s89 20069539

[b20] Dobin A. . STAR: ultrafast universal RNA-seq aligner. Bioinformatics, 29, 15–21 (2013). 23104886 10.1093/bioinformatics/bts635 PMC3530905

[b21] Roberts R. G., Bentley D. R. & Bobrow M. Infidelity in the structure of ectopic transcripts: a novel exon in lymphocyte dystrophin transcripts. Hum. Mutat. 2, 293–299 (1993). 8401537 10.1002/humu.1380020409

[b22] Flanigan K. M. . Nonsense Mutation-Associated Becker Muscular Dystrophy: interplay between exon definition and splicing regulatory elements within the DMD Gene. Hum Mutat 32, 299–308 (2011). 21972111 10.1002/humu.21426 PMC3724403

[b23] Tress M. L. . The implications of alternative splicing in the ENCODE protein complement. Proc Natl Acad Sci USA 104, 5495–5500 (2007). 17372197 10.1073/pnas.0700800104 PMC1838448

[b24] Thanh L. T., Nguyen T. M., Helliwell T. R. & Morris G. E. Characterization of revertant muscle fibers in Duchenne muscular dystrophy, using exon-specific monoclonal antibodies against dystrophin. Am J Hum Genet. 56, 725–731 (1995). 7887428 PMC1801179

[b25] Lu Q. L., Morris G. E., Wilton S. D., Ly T., Artem’yeva O. V., Strong P. & Partridge T. A. Massive idiosyncratic exon skipping corrects the nonsense mutation in dystrophic mouse muscle and produces functional revertant fibers by clonal expansion. J Cell Biol. 148, 985–996 (2000). 10704448 10.1083/jcb.148.5.985 PMC2174546

[b26] Bies R. D. . Human and murine dystrophin mRNA transcripts are differentially expressed during skeletal muscle, heart, and brain development. Nucleic Acids Res. 20, 1725–1731 (1992). 1579466 10.1093/nar/20.7.1725 PMC312263

[b27] Austin R. C., Morris G. E., Howard P. L., Klamut H. J. & Ray P. N. Expression and synthesis of alternatively spliced variants of Dp71 in adult human brain. Neuromuscul Disord. 10, 187–193 (2000). 10734266 10.1016/s0960-8966(99)00105-4

[b28] Ellis J. D. . Tissue-specific alternative splicing remodels protein-protein interaction networks. Mol Cell. 46, 884–892 (2012). 22749401 10.1016/j.molcel.2012.05.037

[b29] Le Rumeur E., Winder S. & Hubert J. F. Dystrophin: More than just the sum of its parts. Biochim Biophys Acta. 1804, 1713–1722 (2010). 20472103 10.1016/j.bbapap.2010.05.001

[b30] Lederfein D. . A 71-kilodalton protein is a major product of the Duchenne muscular dystrophy gene in brain and other nonmuscle tissues. Proc Natl Acad Sci USA 89, 5346–5350 (1992). 1319059 10.1073/pnas.89.12.5346 PMC49288

[b31] Rau F. . Abnormal splicing switch of DMD’s penultimate exon compromises muscle fibre maintenance in myotonic dystrophy. Nat Commun. 6, 7205 (2015). 26018658 10.1038/ncomms8205 PMC4458869

[b32] Nishida A., Minegishi M., Takeuchi A., Awano H., Niba E. T. & Matsuo M. Neuronal SH-SY5Y cells use the C-dystrophin promoter coupled with exon 78 skipping and display multiple patterns of alternative splicing including two intronic insertion events. Hum Genet. 134, 993–1001 (2015). 26152642 10.1007/s00439-015-1581-2

[b33] Irimia M. . A highly conserved program of neuronal microexons is misregulated in autistic brains. Cell 159, 1511–1523 (2014). 25525873 10.1016/j.cell.2014.11.035 PMC4390143

[b34] Li Y., Sanchez-Pulido L., Haerty W. & Ponting C. P. RBFOX and PTBP1 proteins regulate the alternative splicing of micro-exons in human brain transcripts. Genome Res. 25, 1–13 (2015). 10.1101/gr.181990.114 PMC4317164 25524026

[b35] Gazzoli I. . Non-sequential and multi-step splicing of the dystrophin transcript. RNA Biol. 13, 290–305 (2016). 26670121 10.1080/15476286.2015.1125074 PMC4829307

[b36] Koren E., Lev-Maor G. & Ast G. The Emergence of Alternative 3′ and 5′ Splice Site Exons from Constitutive Exons. PLoS Comput Biol. 3, e95 (2007). 17530917 10.1371/journal.pcbi.0030095 PMC1876488

[b37] Hiller M. & Platzer M. Widespread and subtle: alternative splicing at short-distance tandem sites. Trends Gene. 24, 246–255 (2008). 10.1016/j.tig.2008.03.003 18394746

[b38] Yan X., Sablok G., Feng G., Ma J., Zhao H. & Sun X. nagnag: Identification and quantification of NAGNAG alternative splicing using RNA-Seq data. FEBS Lett. 589, 1766–1770 (2015). 26028313 10.1016/j.febslet.2015.05.029

[b39] Ameziane-Le Hir S. . Cholesterol favors the anchorage of human dystrophin repeats 16 to 21 in membrane at physiological surface pressure. Biochim Biophys Acta 1838, 1266–1273 (2014). 24440661 10.1016/j.bbamem.2014.01.010

[b40] Lidov H. G. & Kunkel L. M. Dp140: alternatively spliced isoforms in brain and kidney. Genomics 45, 132–139 (1997). 9339369 10.1006/geno.1997.4905

[b41] Mirabella M., Galluzzi G., Manfredi G., Bertini E., Ricci E., De Leo R., Tonali P. & Servidei S. Giant dystrophin deletion associated with congenital cataract and mild muscular dystrophy. Neurology 51, 592–595 (1998). 9710043 10.1212/wnl.51.2.592

[b42] Blankenberg D. . Galaxy, a web-based genome analysis tool for experimentalists. Curr. Protoc. Mol. Biol. 18, 1199–1216 (2010). 10.1002/0471142727.mb1910s89 PMC4264107 20069535

[b43] Pervouchine D. D., Knowles D. G. & Guigó R. Intron-centric estimation of alternative splicing from RNA-seq data. Bioinformatics 29, 273–274 (2013). 23172860 10.1093/bioinformatics/bts678 PMC3546801

[b44] Langmead B. & Salzberg S. L. Fast gapped-read alignment with Bowtie 2. Nat. Methods 9, 357–359 (2012). 22388286 10.1038/nmeth.1923 PMC3322381

[b45] Kim D. . TopHat2: accurate alignment of transcriptomes in the presence of insertions, deletions and gene fusions. Genome Biol. 14, R36 (2013). 23618408 10.1186/gb-2013-14-4-r36 PMC4053844

[b46] Desmet F. O. . Human Splicing Finder: an online bioinformatics tool to predict splicing signals. Nucleic Acids Res. 37, e67 (2009). 19339519 10.1093/nar/gkp215 PMC2685110

[b47] Yeo G. & Burge C. B. Maximum entropy modeling of short sequence motifs with applications to RNA splicing signals. J. Comput. Biol. 11, 377–394 (2004). 15285897 10.1089/1066527041410418

