# Erratum: Targeted RNA-Seq profiling of splicing pattern in the *DMD* gene: exons are mostly constitutively spliced in human skeletal muscle

**DOI:** 10.1038/srep45414

**Published:** 2017-03-28

**Authors:** Anne-Laure Bougé, Eva Murauer, Emmanuelle Beyne, Julie Miro, Jessica Varilh, Magali Taulan, Michel Koenig, Mireille Claustres, Sylvie Tuffery-Giraud

Scientific Reports 7: Article number: 3909410.1038/srep39094; published online: 01 03 2017; updated: 03 28 2017

The original version of this Article contained errors.

In the Abstract,

“We have analysed the splicing pattern of the human Duchenne Muscular Dystrophy (DMD) NB transcript in normal skeletal muscle”.

now reads:

“We have analysed the splicing pattern of the human Duchenne Muscular Dystrophy (DMD) transcript in normal skeletal muscle”.

In the Discussion section,

“The in-frame skipping of exon 71 results in loss of the syntrophin-binding site from the protein^29^, while the absence of exon 78 causes a frameshiſt that replaces the 13 C-terminal dystrophin amino acids residues with 31 new ones defining a protein with a novel hydrophobic carboxy terminus^30^”.

now reads:

“The in-frame skipping of exon 71 occurs in close proximity to the gene region (exons 73–75) encoding the syntrophin-binding sites^29^, while the absence of exon 78 causes a frameshiſt that replaces the 13 C-terminal dystrophin amino acids residues with 31 new ones defining a protein with a novel hydrophobic carboxy terminus^30^”.

These errors have now been corrected in the PDF and HTML versions of the Article.

