# Supplementary material for: Targeted RNA-Seq profiling of splicing pattern in the DMD gene: exons are mostly constitutively spliced in human skeletal muscle
Source: Sci Rep. 2017 Jan 3;7:39094. doi: 10.1038/srep39094 (PMC5206723; doi:10.1038/srep39094)
Supplement: Supplementary Information [file srep39094-s1.doc]

**Targeted RNA-Seq profiling of splicing pattern in the *DMD* gene: exons are mostly constitutively spliced in human skeletal muscle**

Anne-Laure Bougé^1,2^, Eva Murauer^1,2#^, Emmanuelle Beyne^3^, Julie Miro^1,2^, Jessica Varilh^3^, Magali Taulan^1,2^, Michel Koenig^1,3^, Mireille Claustres^1,2,3^, Sylvie Tuffery-Giraud^1,2^*.

| **Name** | **tissues tested** | **estimated rate of AS** | **Techniques** | **Presence of the ASE** | **References** |
| --- | --- | --- | --- | --- | --- |
| 1a | lymphocytes  human skeletal muscle, heart, lung, prostate | ≈50%-100% in lymphocytes ≤5% in muscle (can be associated with 1b and 1c) | use of specific primers to detect the event in muscle | patient (lymphocytes) and control tissues (heart and skeletal muscle) | (1,2,3) |
| 1b | lymphocytes  human skeletal muscle, heart, lung, prostate | low level | in subcloning sequencing | patient (lymphocytes) and control tissues (heart and skeletal muscle) | (1) |
| 1c | human skeletal muscle, heart, lung, prostate | one clone (associated with 1a and 1b) | (RT)-nested PCRs | control tissues (heart and skeletal muscle) | (1) |
| 2a | skeletal muscle + 12 other tissues (cardiac muscle, brain, stomach, small intestine, colon, lung, spleen, liver, kidney, testis, placenta) | in normal skeletal muscle: very low (associated with del 3-7 and del 3-17) | RT-PCR | skeletal muscle, colon, small intestine (and very low level in cardiac muscle and stomach) | (4) |
| 2b | lymphocytes from patients + 20 normal tissues (adrenal gland, brain cerebellum, whole brain, fetal brain, fetal liver, heart, kidney, liver, whole lung, placenta, prostate, salivary gland, skeletal muscle, spleen, testis, thymus, thyroid gland, trachea, uterus, and spinal cord) | detected in lymphocytes of only 1 /100 patients | (RT)-semi nested PCRs sub-cloning for sequencing | DMD patient carrying a duplication of exon 2 and in normal heart, lung, prostate, salivary gland, and skeletal muscle | (5) |
| 2c-1 | lymphocytes from one patient and normal lymphocytes + skeletal muscle(s?) | very low level and not in lymphocytes of 99 other patients | (RT)-nested PCRs sub-cloning for sequencing | DMD patient carrying a duplication of exons 8 to 11 not in skeletal muscle | (6) |
| 2c-s | lymphocytes from one patient and normal lymphocytes + skeletal muscle | very low level and not in lymphocytes of 99 other patients | (RT)-nested PCRs sub-cloning for sequencing | DMD patient carrying a duplication of exons 8 to 11 not in skeletal muscle | (6) |
| 3a-1 | lymphocytes from one patient and normal lymphocytes + skeletal muscle | very low level and not in lymphocytes of 99 other patients | (RT)-nested PCRs sub-cloning for sequencing | DMD patient carrying a duplication of exons 8 to 11 + normal lymphocytes not in skeletal muscle | (6) |
| 3a-s | lymphocytes from one patient and normal lymphocytes and skeletal muscle | very low level and not in lymphocytes of 99 other patients | (RT)-nested PCRs sub-cloning for sequencing | DMD patient carrying a duplication of exons 8 to 11 not in skeletal muscle | (6) |
| 3a | lymphocytes and skeletal muscle from one patient + 12 different normal tissues (skeletal muscle, cardiac muscle, brain, stomach, small intestine, colon, lung, spleen, liver, kidney, testis, and placenta) | low level in patient's lymphocytes (associated with 1a) | (RT)-nested PCRs | lymphocytes of a patient carrying a point mutation in exon 5, but not in his skeletal muscle. Not present in the 12 additional tissues tested | (3) |
| del2-7 | patient lymphocytes + normal muscle | rare event in normal muscle | RT-PCR and hybridization/radio-active detection | patient carrying a deletion of exons 3-7 in normal muscle | (7,8) |
| del2-9 | patient lymphocytes + normal muscle | rare event in normal muscle | RT-PCR and hybridization/radio-active detection | patient carrying a deletion of exons 3-7 in normal muscle | (7,8) |
| del2-16 | human and mouse muscle samples | low level in both tissues | (RT)-nested PCRs; subcloning for sequencing | normal skeletal muscle | (9) |
| del2-17 | human and mouse muscle samples | very low level in both tissues | (RT)-nested PCRs; subcloning for sequencing | normal skeletal muscle | (9) |
| del3-7 | patient lymphocytes + normal muscle | rare event in normal muscle | RT-PCR and hybridization/  radioactive detection | patient carrying a deletion of exons 3-7 in normal muscle | (7,8) |
| del3-9 | patient lymphocytes + normal muscle | 0.3% of the normal dystrophin transcript in normal muscle (at least 10 times less than in the muscle of patients with the deletion of exons 3-7) | RT-PCR and hybridization/  radioactive detection | patient carrying a deletion of exons 3-7 in normal muscle | (7,8) |
| del3-13 | human and mouse muscle samples | very low level in both tissues | (RT)-nested PCRs; subcloning for sequencing | normal skeletal muscle | (9) |
| del3-16 | human and mouse muscle samples | very low level in both tissues | (RT)-nested PCRs; subcloning for sequencing | normal skeletal muscle | (9) |
| del3-17 | human and mouse muscle samples | very low level in both tissues | (RT)-nested PCRs; subcloning for sequencing | normal skeletal muscle | (9) |
| del4 | normal human muscle (rectum abdomens, gluteus maximum, quadriceps) human cardiac muscle mouse skeletal and cardiac muscle | 100% in rectum abdomens 50% in gluteus maximum | RT-PCR Southern blot | present in some human muscles  absent in quadriceps and mouse tissues | (10) |
| del5-17 | human and mouse muscle samples | very low level in both tissues | (RT)-nested PCRs; subcloning for sequencing | normal skeletal muscle | (9) |
| del9 | human lymphocytes, brain, heart, kidney  mouse liver, lung, skeletal muscle, brain, heart, kidney | consistently detected in non-muscle tissues (50% in lymphocytes) Rare in muscle, heart and brain | RT-PCR | Variable extent in human and mouse tissues not specifically expressing *DMD* 50% in lymphocytes | (11,12) |
| del14+15 | lymphocytes | not determined | RT-nested PCR | carrying a point mutation in exon 24 | (13) |
| 18a | lymphocytes | in one or low number of clones | (RT)-nested PCRs sub-cloning for sequencing | In a patient carrying a splice site mutation in intron 20 | (1) |
| del25 | human lymphocytes and skeletal muscle | not determined | RT-PCR | patient | (14) |
| 29a | lymphocytes | in one or low number of clones | (RT)-nested PCRs sub-cloning for sequencing | patient carrying a mutation in exon 27 | (1) |
| del26-29 | human normal skeletal muscle, heart and brain | present in the three tissues | (RT)-nested PCRs | normal tissues | (15) |
| del26-30 | human normal skeletal muscle, heart and brain | present in the three tissues | (RT)-nested PCRs | normal tissues | (15) |
| del28-29 | human normal skeletal muscle, heart and brain | present in the three tissues | (RT)-nested PCRs | normal tissues | (15) |
| del 35-37 | human normal skeletal muscle, heart and brain | present in the three tissues | (RT)-nested PCRs | normal tissues | (15) |
| del35-42 | human normal skeletal muscle, heart and brain | present in the three tissues | (RT)-nested PCRs | normal tissues | (15) |
| del38 | lymphocytes | present in lymphocytes (undetermined level) | RT-nested PCR | patient | (16) |
| del39 | human normal skeletal muscle, heart and brain  lymphocytes | present in brain and heart | (RT)-nested PCRs | normal tissues and in a patient's lymphocytes carrying a point mutation in exon 59 | (13,15) |
| del41 | human normal skeletal muscle, heart and brain | present in the three tissues | (RT)-nested PCRs | normal tissues | (15) |
| del42 | human normal skeletal muscle, heart and brain | present only in brain and heart | (RT)-nested PCRs | normal tissues | (15) |
| del41-42 | human normal skeletal muscle, heart and brain | present only in brain | (RT)-nested PCRs | normal tissues | (15) |
| del44-56 | human normal skeletal muscle, heart and brain | present in the three tissues | (RT)-nested PCRs | normal tissues | (15) |
| del45-47 | human normal skeletal muscle, heart and brain | present in the three tissues | (RT)-nested PCRs | normal tissues | (15) |
| del45-48 | human normal skeletal muscle, heart and brain | present in the three tissues | (RT)-nested PCRs | normal tissues | (15) |
| del45-49 | human normal skeletal muscle, heart and brain | present only in skeletal muscle and heart | (RT)-nested PCRs | normal tissues | (15) |
| del45-50 | human normal skeletal muscle, heart and brain | present only in skeletal muscle and brain | (RT)-nested PCRs | normal tissues | (15) |
| del45-53 | human normal skeletal muscle, heart and brain | present in the three tissues | (RT)-nested PCRs | normal tissues | (15) |
| del48 | lymphocytes |  | RT-nested PCR | normal control | (13) |
| del49-57 | human normal skeletal muscle, heart and brain | present in the three tissues | (RT)-nested PCRs | normal tissues | (15) |
| del50-51 | fibroblats in one patient and normal muscle | minute amounts in patient's fibroblasts Higher amounts in a normal muscle | Southern blots on cDNA-PCR products | detected in a patient carrying a deletion of exon 50 and in normal muscle | (8) |
| 63a | lymphocytes | in one or low number of clones | (RT)-nested PCRs sub-cloning for sequencing | patient | (1) |
| 67a | lymphocytes | in one or low number of clones | (RT)-nested PCRs sub-cloning for sequencing | patient | (1) |
| del71 | human fetal aorta, brain, heart, leg, stomach lymphocytes | 50% in lymphocytes present in skeletal muscle, brain | nested RT-PCR | tissues | (11,17) |
| del71+ del78 | human fetal aorta, brain, heart, leg, stomach | present in brain | RT-PCR | tissues | (17) |
| del71-72+ del78 | human fetal aorta, brain, heart, leg, stomach | present in brain | RT-PCR | tissues | (17) |
| del71-74 + del78 | human fetal aorta, brain, heart, leg, stomach | present in brain | RT-PCR | tissues | (17) |
| 77a | lymphocytes | in one or low number of clones | (RT)-nested PCRs sub-cloning for sequencing | patient | (1) |
| del 78 | human fetal aorta, brain, heart, leg, stomach | present in skeletal muscle, brain | RT-PCR | tissues | (17) |

**References**

1. Zhang, Z. *et al*. Identification of seven novel cryptic exons embedded in the dystrophin gene and characterization of 14 cryptic dystrophin exons. *J. Hum. Genet*. **52**, 607–17 (2007).
2. Roberts, R. G., Bentley, D. R. & Bobrow, M. Infidelity in the structure of ectopic transcripts: a novel exon in lymphocyte dystrophin transcripts. *Hum. Mutat*. **2**, 293-299 (1993).
3. Suminaga, R. et al. novel cryptic exon in intron 3 of the dystrophin gene was incorporated into dystrophin mRNA with a single nucleotide deletion in exon 5. *J Hum Genet.* **47**, 196-201(2002).
4. [Dwi Pramono, Z. A](http://www.ncbi.nlm.nih.gov/pubmed/?term=Dwi Pramono ZA%5BAuthor%5D&cauthor=true&cauthor_uid=10623618)., [Takeshima, Y](http://www.ncbi.nlm.nih.gov/pubmed/?term=Takeshima Y%5BAuthor%5D&cauthor=true&cauthor_uid=10623618)., [Surono, A](http://www.ncbi.nlm.nih.gov/pubmed/?term=Surono A%5BAuthor%5D&cauthor=true&cauthor_uid=10623618)., [Ishida, T](http://www.ncbi.nlm.nih.gov/pubmed/?term=Ishida T%5BAuthor%5D&cauthor=true&cauthor_uid=10623618). & [Matsuo, M](http://www.ncbi.nlm.nih.gov/pubmed/?term=Matsuo M%5BAuthor%5D&cauthor=true&cauthor_uid=10623618). A novel cryptic exon in intron 2 of the human dystrophin gene evolved from an intron by acquiring consensus sequences for splicing at different stages of anthropoid evolution. *Biochem Biophys Res Commun*. **267**, 321-328 (2000).
5. Tran, V. K. *et al*. A novel cryptic exon identified in the 3' region of intron 2 of the human dystrophin gene. *J Hum Genet*. **50**, 425-33 (2005).
6. Ishibashi, K., Takeshima, Y., Yagi, M., Nishiyama, A. & Matsuo, M. Novel cryptic exons identified in introns 2 and 3 of the human dystrophin gene with duplication of exons 8-11. *Kobe J Med Sci*. **52**, 61-75 (2006).
7. Chelly, J. *et al*. Dystrophin gene transcribed from different promoters in neuronal and glial cells. *Nature* **344**, 64-65 (1990).
8. Chelly, J. *et al*. Illegitimate transcription. Application to the analysis of truncated transcripts of the dystrophin gene in nonmuscle cultured cells from Duchenne and Becker patients. *J Clin Invest*. **88**, 1161-1166 (1991).
9. Surono, A., Takeshima, Y., Wibawa, T., Pramono, Z. A. & Matsuo, M. Six novel transcripts that remove a huge intron ranging from 250 to 800 kb are produced by alternative splicing of the 5’ region of the dystrophin gene in human skeletal muscle. *Biochem. Biophys. Res. Commun*. **239**, 895–899 (1997).
10. Torelli, S. & Muntoni, F. Alternative splicing of dystrophin exon 4 in normal human muscle. *Hum Genet*. **97**, 521-523 (1996).
11. Reiss, J. & Rininsland, F. An explanation for the constitutive exon 9 cassette splicing of the DMD gene. *Hum Mol Genet*. **3**, 295-298 (1994).
12. Roberts, R. G., Barby, T. F., Manners, E., Bobrow, M. & Bentley, D.R. Direct detection of dystrophin gene rearrangements by analysis of dystrophin mRNA in peripheral blood lymphocytes. *Am J Hum Genet*. **49**, 298-310 (1991).
13. Tuffery, S., Bareil, C., Demaille, J. & Claustres, M. Four novel dystrophin point mutations: detection by protein truncation test and transcript analysis in lymphocytes from Duchenne muscular dystrophy patients. *Eur J Hum Genet*. **4**, 143-152 (1996).
14. Barbieri, A.M. *et al*. Seven novel additional small mutations and a new alternative splicing in the human dystrophin gene detected by heteroduplex analysis and restricted RT-PCR heteroduplex analysis of illegitimate transcripts. *Eur J Hum Genet*. **4**, 183-187 (1996).
15. Sironi, M. *et al*. The dystrophin gene is alternatively spliced throughout its coding sequence. *FEBS Lett*. **517**, 163-166 (2002).

16. [Gardner, R.J](http://www.ncbi.nlm.nih.gov/pubmed/?term=Gardner RJ%5BAuthor%5D&cauthor=true&cauthor_uid=7668256)., [Bobrow, M](http://www.ncbi.nlm.nih.gov/pubmed/?term=Bobrow M%5BAuthor%5D&cauthor=true&cauthor_uid=7668256). & [Roberts, R. G](http://www.ncbi.nlm.nih.gov/pubmed/?term=Roberts RG%5BAuthor%5D&cauthor=true&cauthor_uid=7668256). The identification of point mutations in Duchenne muscular dystrophy patients by using reverse-transcription PCR and the protein truncation test. *Am J Hum Genet*. **57**, 311-320 (1995).

17. Feener, C. A., Koenig, M. & Kunkel, M. Alternative splicing of human dystrophin mRNA generates isoforms at the carboxy terminus. *Nature*, **338**, 509-511 (1989).
